# Examining the relationship between problematic social media use and caffeine use disorder in the context of physical activity: a cross-sectional moderation study among university students

**DOI:** 10.3389/fnut.2026.1854577

**Published:** 2026-06-08

**Authors:** Bekir Erhan Orhan, Buket Karadağ, Walaa Jumah Alkasasbeh, Adam Tawfiq Amawi

**Affiliations:** 1 Faculty of Sports Sciences, Istanbul Aydın University, Istanbul, Türkiye; 2 Faculty of Education, Istanbul Aydın University, Istanbul, Türkiye; 3 Department of Physical Education, School of Sports Sciences, The University of Jordan, Amman, Jordan; 4 Department of Movement Sciences and Sports Training, School of Sports Sciences, The University of Jordan, Jordan

**Keywords:** behavioural addiction, caffeine use disorder, physical activity, problematic social media use, social media addiction, university students

## Abstract

**Introduction:**

Problematic social media use (PSMU) and caffeine use disorder (CUD), a behavioral pattern without formal diagnostic status and a DSM-5 condition listed for further study, respectively, share phenomenological features (tolerance, withdrawal, compulsive engagement) that make their statistical co-occurrence among young adults theoretically plausible and warrant joint empirical examination. Yet the potential moderating role of lifestyle factors such as physical activity remains largely unexplored. This study examined the relationship between problematic social media use, hereafter termed social media addiction (SMA), and caffeine use disorder (CUD) among university students, and determined whether physical activity (PA) level moderates this relationship.

**Methods:**

A cross-sectional correlational survey design was employed. Data were collected online from 408 Turkish university students (59.1% women; *M* = 22.38 years, *SD* = 2.14) using the Bergen Social Media Addiction Scale (BSMAS), the Caffeine Use Disorder Questionnaire (CUDQ), and the International Physical Activity Questionnaire-Short Form (IPAQ-SF). Pearson correlation, one-way ANOVA with Tukey HSD and Games-Howell *post hoc* comparisons, and hierarchical moderated regression were employed.

**Results:**

A statistically significant small-to-moderate positive correlation emerged between SMA and CUD (*r* = 0.374, *p* < 0.001). SMA scores did not differ significantly across PA groups (inactive, minimally active, sufficiently active), *F* (2, 405) = 0.177, *p* = 0.838, *η*^2^*p* = 0.001. CUD scores, however, differed significantly by PA level, *F* (2, 405) = 4.458, *p* = 0.012, *η*^2^*p* = 0.021, with inactive students reporting lower CUD than both active groups, a pattern that is descriptively compatible with, but does not directly test, the ergogenic caffeine use hypothesis. Physical activity did not moderate the SMA-CUD relationship (*β* = 0.043, *p* = 0.358; Δ*R*^2^ = 0.002), although SMA remained a significant direct predictor of CUD (*β* = 0.373, *p* < 0.001).

**Discussion:**

These cross-sectional findings suggest that PSMU and CUD co-occur to a modest but detectable degree among university students, and that habitual PA neither attenuates nor amplifies this association. Given the modest effect sizes and the design, implications for screening and intervention are discussed cautiously and treated as hypothesis-generating for prospective and experimental work.

## Introduction

The proliferation of digital technologies and the parallel growth in caffeinated product consumption have drawn empirical attention to two patterns of high-frequency engagement among young adults: problematic social media use (PSMU) and caffeine use disorder (CUD), the latter currently listed in the DSM-5 as a condition warranting further study rather than as an established disorder (1, 2). PSMU is conceptualised within the behavioural addiction framework, and the term “social media addiction” (SMA) is used hereafter only as a shorthand for high-severity, addiction-like engagement consistent with prior literature; it does not imply formal diagnostic status, since neither the DSM-5 nor the ICD-11 currently recognises social media use as a disorder, and the field continues to debate whether an addiction model is the most appropriate framing (3, 4). By contrast, CUD is codified under DSM-5 research criteria as a substance-related disorder warranting further investigation (5, 6). Despite this nosological asymmetry, both patterns share key phenomenological and neurobiological features, including tolerance, withdrawal, compulsive use, and dopaminergic reward pathway involvement, making their co-occurrence theoretically plausible and empirically tractable (4, 7, 8). University students constitute a population with elevated exposure to risk conditions for both behaviours, rather than a clinically high-risk group per se: the transition to higher education is characterised by heightened autonomy over lifestyle choices, intensified academic and social stressors, disrupted sleep, and amplified social comparison pressures, conditions that collectively promote the emergence and entrenchment of problematic behavioural patterns (9–11). Although these two literatures each provide candidate mechanisms for the SMA-CUD link (shared dopaminergic vulnerability, transdiagnostic dispositional risk, and the sleep-caffeine pathway), the empirical question of whether PSMU and CUD scores actually co-vary within the same individuals, and whether this co-variation is modified by lifestyle factors such as physical activity (PA), has rarely been tested directly. The present study provides such a direct empirical test rather than a new mechanistic account.

SMA is characterised within the behavioural addiction framework by salience, mood modification, tolerance, withdrawal, conflict, and repeated unsuccessful attempts to curtail use (3, 4). Platform architectures, engineered around variable-ratio reinforcement schedules through notifications, likes, and social feedback loops, have substantially elevated the addictive potential of digital social environments (3). Epidemiological evidence indicates that problematic social media use affects between 5 and 10% of young adult users globally, with prevalence estimates varying depending on operational definitions and classification criteria (2, 12). University students are disproportionately exposed to risk factors for SMA. Fear of missing out (FoMO), a pervasive apprehension that peers are experiencing rewarding events in one’s absence, is a robust predictor of compulsive social media checking, mediated by social comparison processes and self-esteem deficits (13–15). Unmet needs for social belonging, identity exploration, and management of academic-social tensions, situated within a self-determination framework, further amplify vulnerability to high-frequency problematic use (16). Elevated SMA is associated with delayed sleep onset, reduced sleep duration, poorer sleep quality, anxiety, depression, and diminished subjective well-being (10, 17). The neurobiological substrate of addiction-like social media use overlaps substantially with that of pharmacological addictions: social media stimuli activate mesolimbic dopaminergic pathways through positive and negative reinforcement mechanisms, producing reward signals that sustain compulsive engagement despite adverse consequences, a profile that has led researchers to frame high-level problematic use in addiction terms, even absent formal diagnostic codification (4, 8).

Caffeine is the world’s most widely consumed psychoactive substance, with habitual use well documented within university student culture through coffee, energy drinks, and pre-workout formulations (18, 19). Caffeine exerts its primary psychoactive effects through adenosine receptor antagonism, with downstream facilitation of dopaminergic, noradrenergic, and cholinergic signalling, enhancing alertness and attenuating fatigue (7). Habitual high-dose consumption, however, is associated with the development of tolerance, withdrawal symptoms upon cessation, including headache, fatigue, irritability, and impaired concentration, and clinically significant functional impairment, collectively operationalised as CUD under DSM-5 research criteria (1, 20). Academic performance pressures, extended study schedules, chronic sleep debt, and the social normalisation of caffeinated beverages collectively drive high-frequency use, with prevalence estimates for CUD among university students ranging from approximately 7 to 15% across studied samples (1, 20, 21). Caffeine’s rewarding properties also overlap neurobiologically with those implicated in other addictive patterns: indirect facilitation of dopamine release in mesolimbic reward circuits produces motivationally significant states that may interact with vulnerability factors common across addictive behaviours (7, 19).

These literatures can be integrated under Brand and colleagues’ Interaction of Person-Affect-Cognition-Execution (I-PACE) model of addictive behaviours (22), which posits that core person variables (e.g., reward sensitivity, impulsivity, emotion dysregulation) interact with affective and cognitive responses to specific stimuli (notifications, caffeine cues) to produce conditioned, reinforcing engagement that becomes increasingly automatised. Within the I-PACE architecture, the co-occurrence of PSMU and CUD is anticipated whenever the same person variables sensitise the individual to multiple reinforcing stimuli; against this common scaffold, three more specific and analytically separable explanatory frameworks have been advanced. First, shared neurobiological vulnerability, specifically heightened mesolimbic dopaminergic reactivity and deficient prefrontal inhibitory control, may render the same individuals susceptible to multiple addictive patterns simultaneously (4, 8). Second, transdiagnostic psychological dispositions, including trait impulsivity, emotion dysregulation, and reward hypersensitivity, are robust cross-cutting predictors of both behavioural and substance-related addictions and thus constitute candidate common aetiological pathways for co-occurring disorders (9, 23, 24). Third, a direct behavioural mechanism is plausible: protracted nocturnal social media use disrupts sleep architecture, generating daytime fatigue that motivates compensatory caffeine consumption and sustains a mutually reinforcing cyclical relationship (10, 17).

PA has been proposed as a potentially protective factor against a range of mental health difficulties and addictive behaviours, with regular exercise conferring neurobiological adaptations, including enhanced prefrontal cortical function, modulation of dopaminergic tone, and attenuation of stress-induced HPA-axis reactivity, that may broadly reduce vulnerability to addictive engagement (25, 26). Evidence suggests inverse relationships between habitual PA and various problematic technology use behaviours, with some data indicating that active individuals may be partially buffered against high-level addiction-like social media engagement (2, 27). However, the relationship between PA and CUD introduces theoretical complexity: caffeine is among the most extensively documented ergogenic aids in exercise science, with well-established acute benefits for endurance performance, strength output, and attentional focus (18, 28). This raises the theoretically grounded possibility that physically active students may not exhibit lower CUD severity; rather, performance-oriented ergogenic use may actually elevate disorder-level consumption in this subgroup. Two competing predictions therefore arise: a buffering hypothesis, in which higher PA levels weaken the SMA-CUD coupling because exercise-related neurobiological adaptations and lifestyle structure attenuate shared vulnerability mechanisms; and an exposure hypothesis, in which higher PA levels strengthen or leave unchanged the SMA-CUD coupling because active students are simultaneously embedded in a performance-oriented caffeine culture. No directional moderation hypothesis is advanced here; rather, the moderation test is framed as an empirical adjudication between these competing accounts, which constitutes the primary contribution of the present study to the integrative I-PACE-informed framework outlined above.

The present study therefore examined the relationship between SMA and CUD among university students and tested whether PA level moderates this association. Five research questions guided the inquiry: (1) What are the levels of SMA, CUD, and PA among university students? (2) Is there a statistically significant relationship between SMA and CUD? (3) Do SMA scores differ significantly according to PA level? (4) Do CUD scores differ significantly according to PA level, with physically active students expected to report higher CUD severity consistent with the ergogenic caffeine use hypothesis? (5) Does PA level play a moderating role in the SMA-CUD relationship?

## Materials and methods

### Research design

This study employed a quantitative cross-sectional correlational survey design to examine the association between social media addiction and caffeine use disorder among university students and to determine whether this association varied as a function of physical activity level. Such designs are specifically suited to identifying and characterising the co-variation among variables without experimental manipulation (29). Given the convenience sampling procedure employed, findings should be interpreted within the bounds of the recruited sample rather than generalised to the broader university student population.

### Participants

Participants were recruited via convenience sampling through an online self-administered survey distributed via institutional communication channels. Although this approach does not guarantee equal selection probability across all members of the target population, it is consistent with established practice in cross-sectional survey research within university settings (30). The initial sample comprised 441 volunteering university students. Before analysis, missing-value and multivariate-outlier screening procedures (Mahalanobis distance, *p* < 0.001) (31) were applied; 33 records were consequently excluded, yielding a final analytic sample of *N* = 408. Of these, 59.1% were women (*n* = 241) and 40.9% were men (*n* = 167). Academic-year distribution was as follows: 30.6% were first-year students (*n* = 125), 28.2% were second-year students (*n* = 115), 10.8% were third-year students (*n* = 44), and 30.4% were fourth-year students (*n* = 124). The mean age of the sample was 22.38 years (SD = 2.14). Body mass index, calculated from self-reported height and weight, had a mean of 23.32 (SD = 4.80), indicating that participants, on average, fell within the normal weight range.

### Measures

#### Bergen social media addiction scale

The BSMAS was developed by Andreassen et al. (32) to assess addiction-like engagement with social media platforms. Its Turkish adaptation was validated by Demirci (33). The scale consists of six items, each representing a core component of behavioural addiction: salience, mood modification, tolerance, withdrawal, conflict, and relapse. Items are rated on a five-point Likert scale ranging from 1 (Very rarely) to 5 (Very often), yielding total scores between 6 and 30; higher scores indicate greater addiction severity. The original scale demonstrated a Cronbach’s alpha of 0.88; internal consistency in the present study was *α* = 0.83, indicating acceptable reliability. Although no universally validated cut-off score has been established for the BSMAS, a total score of ≥ 18 (corresponding to a mean item response of ≥ 3 across all six components) has been used as a summary threshold for elevated addiction risk in descriptive comparisons (2, 32). Descriptive findings are interpreted using this total-score threshold, consistent with the scale’s continuous scoring properties and the parametric analyses employed.

#### Caffeine use disorder questionnaire

The CUDQ was developed by Ágoston et al. (5) to operationalise the nine criteria for caffeine use disorder proposed in DSM-5 as a condition for further study; CUDQ scores therefore index the severity of research-criteria CUD symptomatology rather than a formally established psychiatric diagnosis. The questionnaire comprises 10 items rated on a four-point scale (1 = Never to 4 = Very often), yielding total scores ranging from 10 to 40; higher scores reflect greater disorder severity. Participants reported symptoms experienced over the preceding 12 months. The Turkish psychometric validation (34) yielded Cronbach’s alpha = 0.86 and an intraclass correlation coefficient of 0.83. In the present study, internal consistency was *α* = 0.94, reflecting excellent reliability.

#### International physical activity questionnaire-short form

Physical activity was assessed using the IPAQ-SF, a standardised self-report instrument for quantifying weekly physical activity in adults (35). The short form consists of seven items covering vigorous-intensity activities, moderate-intensity activities, walking, and sedentary behaviour over the past 7 days; only bouts of at least 10 consecutive minutes are counted. Activity volume is expressed in metabolic equivalent task minutes per week (MET-min/week) using standard coefficients: walking = 3.3 MET, moderate activity = 4.0 MET, and vigorous activity = 8.0 MET. Participants were classified into three groups using the IPAQ-SF official scoring protocol (35): inactive (< 600 MET-min/week), minimally active (600–3,000 MET-min/week), and sufficiently active (> 3,000 MET-min/week). This three-level categorisation was retained for descriptive and ANOVA-based group comparisons because it preserves the IPAQ-SF’s internationally standardised cut-points and supports interpretability against the existing population-level PA literature; the moderated-regression analysis, however, used the continuous MET-min/week metric to maximise statistical power and avoid the information loss associated with discretisation (36). Turkish validity and reliability were established by Öztürk (37) and Sağlam et al. (38). Although this reliability estimate falls marginally below the conventional threshold of 0.70, the IPAQ-SF retains established international validity (35) and is widely used in population-level physical activity research. As a self-report instrument, the IPAQ-SF may be susceptible to recall and social desirability biases.

### Procedure

Data collection was conducted online. The survey comprised a personal information form followed by the three scales described above. Participants received a full description of the study’s purpose and procedures at the outset; electronic informed consent was obtained from all participants before they completed the scales. Participation was entirely voluntary and anonymous.

### Data analysis

All analyses were performed using SPSS version 24 (IBM Corp., 2016). Distributional normality was assessed using skewness and kurtosis indices, supplemented by visual inspection of Q-Q plots. Given the sample size (*N* > 200), the skewness-kurtosis criterion was prioritised over formal significance tests, consistent with the recommendations of Field (39) and Tabachnick and Fidell (31). All three variables fell within the acceptable range of ±1 (see Table 1), confirming approximate normality and supporting the use of parametric procedures.

**Table 1:** Normality statistics for study variables.

Variable	*n*	Min	Max	Skewness	Kurtosis
SMA	408	6	30	0.106	−0.311
CUD	408	10	40	0.477	−0.711
PA (MET-min/week)	408	< 600	6,491	0.359	−0.364

SMA, Social Media Addiction; CUD, Caffeine Use Disorder; PA, Physical Activity. All skewness and kurtosis values fall within ±1, confirming approximate normality.

Pearson product–moment correlation was used to examine the bivariate association between SMA and CUD. One-way analyses of variance (ANOVAs) were conducted to test whether SMA and CUD scores differed across the three PA groups. Homogeneity of variance was assessed using Levene’s test; where the assumption was violated, the Games-Howell *post hoc* test was applied. The moderating role of PA level in the SMA-CUD relationship was evaluated using hierarchical moderated regression, with sex entered as a covariate in Step 1 alongside SMA and PA, and the interaction term (SMA × PA) added in Step 2. Both continuous predictors were mean-centred before computing the interaction term to reduce non-essential multicollinearity (40). Multicollinearity was assessed using variance inflation factors (VIFs) and tolerance values; all VIFs were below 1.05, and all tolerance values exceeded 0.50.

Preliminary independent-samples *t*-tests and chi-square analyses confirmed that SMA scores did not differ significantly by sex [*t* (406) = 1.21, *p* = 0.227] and that the sex distribution across PA groups was proportionate [*χ*^2^ (2) = 2.14, *p* = 0.343]. Sex was nonetheless retained as a covariate in all regression models given its marginally significant association with CUD (*β* = 0.092, *p* = 0.067) and the notable sex imbalance in the present sample (59.1% women). An *a priori* power analysis (G*Power 3.1) (41) indicated a minimum sample of *N* = 395 to detect a small incremental interaction effect (*f*^2^ = 0.02, based on the *R*^2^ increment at Step 2) with power of 1 − *β* = 0.80 and *α* = 0.05; the achieved sample (*N* = 408) marginally exceeded this threshold.

### Ethical approval

This study was conducted in accordance with the Higher Education Institutions’ Scientific Research and Publication Ethics Directive. All procedures were approved by the Ethics Committee of Istanbul Aydın University (Decision No: 2026/02).

## Results

**Figure 1:**
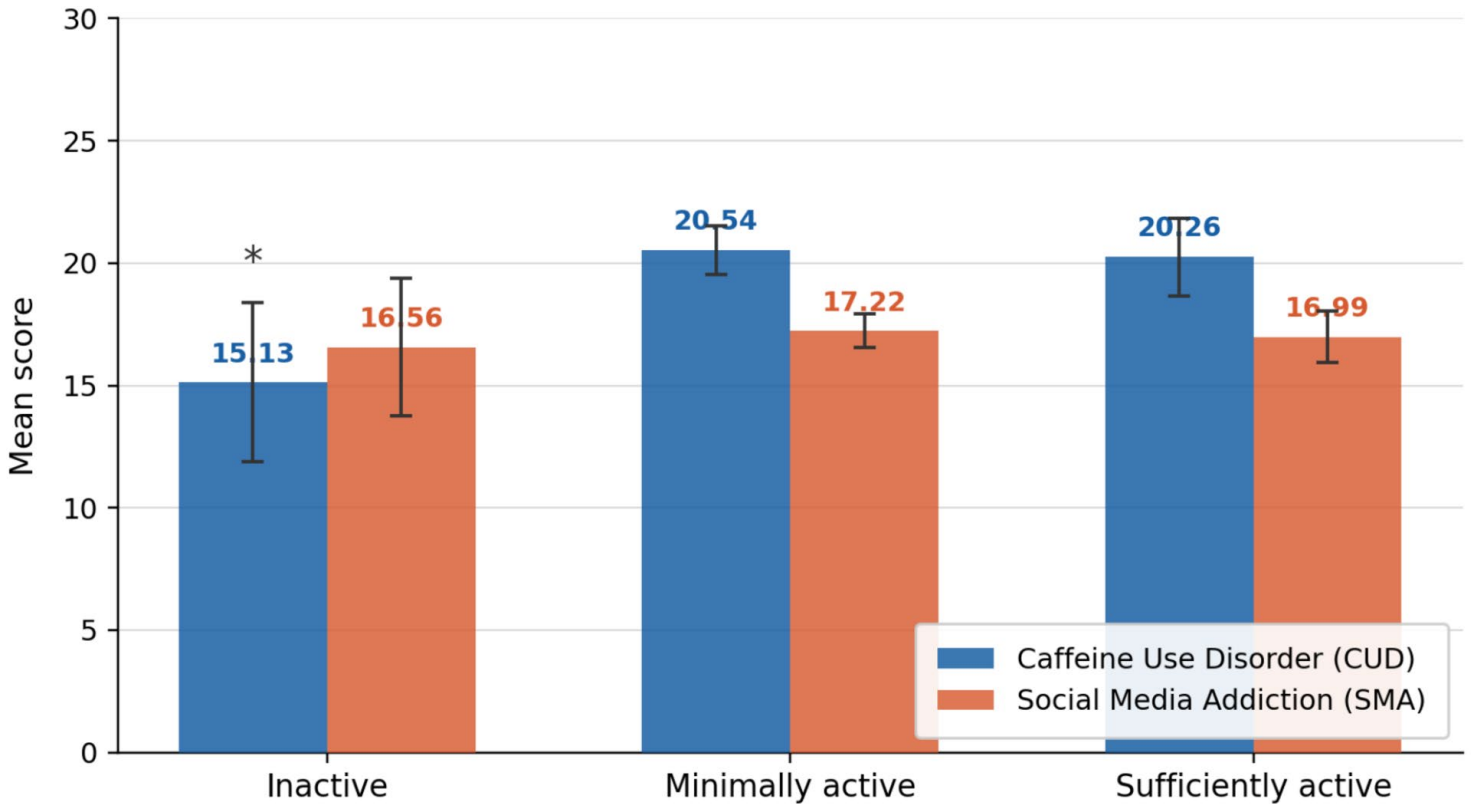
Mean CUD and SMA scores by PA group with 95% confidence intervals. Inactive: *n* = 23; minimally active: *n* = 257; sufficiently active: *n* = 128. *Inactive group differs significantly from both active groups for CUD (*p* = 0.012); no significant group differences for SMA (*p* = 0.838). Error bars represent 95% CIs. CUD, Caffeine Use Disorder; SMA, Social Media Addiction; PA, Physical Activity.

Descriptive statistics and normality indices are presented in Tables 1, 2, with group-level descriptive data by PA stratum in Table 3. Results of correlation, ANOVA, and moderated regression analyses are presented in Tables 4–7, and Figure 1 provides a graphical summary of group-level CUD and SMA means.

**Table 2:** Descriptive statistics for study variables.

Variable	*n*	*M*	SD	Possible/Observed range
SMA	408	17.11	5.77	6–30
CUD	408	20.15	8.41	10–40
PA (MET-min/week)	408	2,430.10	1,201.86	< 600–6,491

SMA, Social Media Addiction; CUD, Caffeine Use Disorder; PA, Physical Activity.

**Table 3:** Descriptive statistics for CUD and SMA by PA group.

Variable	PA group	*n*	*M*	SD	Min	Max	95% CI [LL, UL]
CUD	Inactive	23	15.13	7.50	10	40	[11.89, 18.38]
	Minimally active	257	20.54	8.03	10	40	[19.55, 21.53]
	Sufficiently active	128	20.26	9.07	10	40	[18.68, 21.85]
	Total	408	20.15	8.41	10	40	[19.33, 20.97]
SMA	Inactive	23	16.57	6.51	6	30	[13.75, 19.38]
	Minimally active	257	17.22	5.59	6	30	[16.54, 17.91]
	Sufficiently active	128	16.99	6.02	6	30	[15.94, 18.04]
	Total	408	17.11	5.77	6	30	[16.55, 17.67]

PA, physical activity. Group classifications follow IPAQ-SF criteria: Inactive < 600 MET-min/week; Minimally Active = 600–3,000 MET-min/week; Sufficiently Active > 3,000 MET-min/week. 95% CI = 95% confidence interval for the mean [LL, lower limit; UL, upper limit]. CUD, Caffeine Use Disorder; SMA, Social Media Addiction. Inactive group was markedly underrepresented (*n* = 23, 5.6%); interpret between-group comparisons accordingly.

**Table 4:** Pearson correlation between SMA and CUD.

Variable pair	*N*	*r*	*p*	95% CI [LL, UL]
Social media addiction-caffeine use disorder	408	0.374***	< 0.001	[0.282, 0.460]

*N* = 408. 95% CI computed using Fisher’s r-to-z transformation. ***p < 0.001.

**Table 5:** One-way ANOVA results for SMA by PA level.

Source	SS	df	MS	F	*p*	*η* ^2^ *p*
Between groups	11.81	2	5.91	0.177	0.838	0.001
Within groups	13,517.00	405	33.38	–	–	–
Total	13,528.81	407	–	–	–	–

*η*^2^*p* = η^2^ = SSbetween/SStotal = 11.81/13,528.81 = 0.001 (for one-way ANOVA, partial *η*^2^ equals *η*^2^). Levene’s test of homogeneity of variance was non-significant, *F* (2, 405) = 1.60, *p* = 0.203; Tukey HSD post hoc comparisons were applied but yielded no significant pairwise differences.

**Table 6:** One-way ANOVA results for CUD by PA level.

Source	SS	df	MS	*F*	*p*	*η* ^2^ *p*
Between groups	620.48	2	310.24	4.458	0.012*	0.021
Within groups	28,187.40	405	69.60	–	–	–
Total	28,807.88	407	–	–	–	–

*η*^2^*p* = 0.021. Levene’s test indicated heterogeneity of variance, *F* (2, 405) = 3.18, *p* = 0.042; the Games-Howell post hoc procedure was therefore applied. Significant pairwise differences: Inactive vs. Minimally Active (*p* = 0.008); Inactive vs. Sufficiently Active (*p* = 0.016). No significant difference between Minimally Active and Sufficiently Active (*p* = 0.954). *p < 0.05.

**Table 7:** Hierarchical moderated regression results for the role of PA in the SMA-CUD relationship.

Variable	*B*	SE B	*β*	*t*	*p*	VIF
Step 1						
Sex (covariate)	0.793	0.432	0.092	1.836	0.067	1.01
SMA (×)	0.544	0.067	0.373	8.083	< 0.001	1.02
PA (M)	0.000	0.000	0.007	0.152	0.879	1.01
Step 2						
Sex (covariate)	0.794	0.432	0.092	1.836	0.067	1.01
SMA (×)	0.545	0.067	0.373	8.101	< 0.001	1.02
PA (M)	0.000	0.000	0.007	0.152	0.879	1.03
Interaction (X × M)	4.80 × 10^−5^	0.000	0.043	0.921	0.358	1.04

Dependent variable: Caffeine Use Disorder. Both SMA and PA were mean-centred prior to interaction term computation. Step 1: *R*^2^ = 0.142, *F* (3, 404) = 22.36, *p* < 0.001. Step 2: *R*^2^ = 0.144, Δ*R*^2^ = 0.002, Δ*F* (1, 403) = 0.848, *p* = 0.358. Sex was coded 0 = male, 1 = female. VIF = variance inflation factor; all VIF values < 1.05. *β* = standardised coefficient. The unstandardised coefficient for the interaction term (*B* = 4.80 × 10^−5^) is very small because PA is expressed in raw MET-min/week units (range: hundreds to thousands); the standardised coefficient (*β* = 0.043) provides the appropriate scale-free effect size for interpretation.

### Distributional properties and descriptive statistics

As shown in Table 1, all skewness and kurtosis values fell within the ±1 range, confirming that the distributional assumptions required for parametric analyses were satisfied. The mean BSMAS score was 17.11 (SD = 5.77). The sample mean fell just below the previously reported summary cut-off of ≥ 18 used descriptively in BSMAS-based literature (2, 32). Because no validated clinical referent exists for this cut-off, it is reported here for descriptive comparability only and is not interpreted diagnostically or used to define a high-risk subgroup. The mean CUDQ score was 20.15 (SD = 8.41), indicating moderate CUD severity within the possible range of 10–40 (34). The mean PA score was 2,430.10 MET-min/week (SD = 1,201.86), placing participants on average within the minimally active category (600–3,000 MET-min/week), though notably close to the sufficiently active threshold (≥ 3,000 MET-min/week).

As shown in Table 3 and Figure 1, CUD scores were markedly lower in the inactive group (*M* = 15.13, SD = 7.50) relative to both the minimally active (*M* = 20.54, SD = 8.03) and sufficiently active (*M* = 20.26, SD = 9.07) groups. SMA scores displayed negligible variation across PA groups (inactive: *M* = 16.57, SD = 6.51; minimally active: *M* = 17.22, SD = 5.58; sufficiently active: *M* = 16.99, SD = 6.01), with overlapping confidence intervals across strata.

### Relationship between SMA and CUD

As shown in Table 4, there was a statistically significant, positive, small-to-moderate correlation between SMA and CUD [*r* = 0.374, *p* < 0.001, 95% CI (0.282, 0.460)]. Higher SMA scores were therefore associated with higher CUD scores in the present sample; the magnitude of this association is small-to-moderate (42), and the cross-sectional design precludes any inference about temporal precedence or direction of effect.

### SMA scores by PA level

The one-way ANOVA revealed no statistically significant difference in SMA scores across PA groups, *F* (2, 405) = 0.177, *p* = 0.838 (see Table 5). The effect size was negligible (*η*^2^*p* = 0.001), confirming the practical absence of any meaningful association between PA level and SMA severity.

### CUD scores by PA level

CUD scores differed significantly across PA groups, *F* (2, 405) = 4.458, *p* = 0.012 (see Table 6). The effect size was small (*η*^2^*p* = 0.021), indicating that PA level accounted for approximately 2% of the variance in CUD scores. Games-Howell *post hoc* comparisons revealed that inactive students reported significantly lower CUD than both minimally active and sufficiently active students. No significant difference was found between the two active groups. Because the inactive subgroup was small (*n* = 23, 5.6%), the inactive-vs.-active contrasts (*p* = 0.008; *p* = 0.016) and the apparent ANOVA-regression discrepancy are revisited cautiously in the Discussion; any threshold or non-linear interpretation requires formal testing in larger, balanced samples and is not advanced here.

### Moderating role of PA in the SMA-CUD relationship

The full regression model at Step 1 (sex, SMA, and PA as predictors) explained 14.2% of the variance in CUD [*R*^2^ = 0.142, *F* (3, 404) = 22.36, *p* < 0.001; see Table 7]. The addition of the interaction term (SMA × PA) in Step 2 yielded an increment of Δ*R*^2^ = 0.002, which was not statistically significant [Δ*F* (1, 403) = 0.848, *p* = 0.358], indicating that PA did not moderate the SMA-CUD relationship. The direct effect of SMA on CUD remained statistically significant and stable across both steps (*β* = 0.373, *p* < 0.001). In contrast, PA was not a significant predictor of CUD when included alongside SMA and sex (*β* = 0.007, *p* = 0.879). Multicollinearity diagnostics confirmed that the non-significant interaction term is not due to collinearity: VIF values for all predictors ranged from 1.01 to 1.04, well below the conventional threshold of 5.0 (39).

## Discussion

This study examined the association between SMA and CUD among university students and tested whether PA level moderated this relationship. Given the cross-sectional design, all mechanistic and directional interpretations offered below are explicitly speculative and are intended to generate hypotheses for prospective and experimental investigation rather than to establish causal relationships.

### SMA and CUD

The first and theoretically most central finding was the statistically significant, small-to-moderate positive correlation between SMA and CUD (*r* = 0.374, *p* < 0.001), indicating that greater SMA severity co-occurs with greater CUD severity. This result is consistent with the broader behavioural co-occurrence literature, which documents convergent addiction-like behavioural and substance-related patterns within the same individuals (3, 23, 24). Importantly, SMA and CUD occupy asymmetric nosological positions: CUD carries formal DSM-5 research-criteria status as a substance-related disorder, while SMA, as operationalised by the BSMAS, reflects a theoretically grounded addiction-like behavioural pattern that has not yet attained formal diagnostic recognition. This distinction does not invalidate the observed association, but it does require that findings be interpreted as documenting co-occurring risk patterns rather than comorbid clinical diagnoses in the strict psychiatric sense.

At a theoretical level, both PSMU and CUD have been described as engaging reward-related processes (4, 7); however, no neurobiological variables were measured in the present study, and any specific neural-mechanism account is therefore offered only as one of several candidate explanations to be tested in future work using imaging, pharmacological, or biomarker-based designs. The observed correlation is accordingly not interpreted here as evidence for any particular neural pathway. A plausible behavioural mechanism linking SMA and CUD involves disrupted sleep architecture. Excessive nocturnal social media use has been cross-sectionally associated with delayed sleep onset, reduced sleep duration, and poorer sleep quality (10, 17, 43, 44). Sleep disruption of this kind plausibly motivates compensatory caffeine consumption as a wakefulness strategy, thereby sustaining a mutually reinforcing cycle between the two behavioural patterns. This directional pathway cannot be established from the present cross-sectional data; prospective and experimental designs will be required to test this proposed sequence. Critically, of the three explanatory frameworks outlined in the Introduction, shared neurobiological vulnerability, transdiagnostic psychological dispositions, and the sleep-disruption pathway, this last mechanism offers the most directly testable causal chain linking SMA to CUD, because it specifies a sequential, temporally ordered process amenable to prospective diary, ecological momentary assessment, and actigraphy-based designs. Establishing or disconfirming this pathway should therefore constitute a priority for future research. Transdiagnostic vulnerability factors, including impulsivity, emotion dysregulation, and reward hypersensitivity, have been identified as candidate common aetiological pathways for co-occurring addictive behaviours (9, 45), though their specific role in the SMA-CUD relationship observed here remains empirically unexamined. The sample means for SMA (*M* = 17.11) and CUD (*M* = 20.15) place a non-trivial proportion of participants in the upper range of both measures. Beyond neurobiological accounts, several psychological and contextual factors plausibly contribute to this co-occurrence: examination-period workload, performance-driven study cultures, social-comparison and FoMO-related distress, family and peer norms surrounding caffeine consumption, and broader sociocultural pressures around academic productivity and digital connectedness all merit explicit consideration alongside any neurobiological interpretation. None of these factors was directly measured here, and they should be modelled jointly with biological indices in future research.

### SMA across PA groups

The second finding was that SMA scores did not differ significantly across PA groups, *F* (2, 405) = 0.177, *p* = 0.838. This null result indicates that no statistically significant association between habitual PA level and SMA severity was detected in the present sample. Given the markedly underrepresented inactive group (*n* = 23, *η*^2^*p* = 0.001), this finding warrants careful qualification: the present study did not detect a statistically meaningful PA-SMA association, rather than establishing the absence of such an association. Given the near-zero effect size (*η*^2^*p* = 0.001), the most parsimonious interpretation is that SMA and habitual PA are largely independent constructs in the present sample. This independence is broadly consistent with the view that the motivational antecedents of problematic social media use, including fear of missing out, social comparison tendencies, and the pursuit of social validation, are primarily psychological rather than lifestyle-behavioural in nature (13–15, 27), and that the ubiquity of smartphone access renders social media engagement structurally independent of sedentary versus active contexts (25). However, extensive theoretical rationalisation of an effect this small risks overinterpretation; the finding is best treated as indicating that PA level, as classified by the IPAQ-SF, does not meaningfully differentiate students in terms of SMA severity.

### CUD across PA groups

Consistent with the directional hypothesis advanced in the Introduction, inactive students exhibited significantly lower CUD scores than both minimally active and sufficiently active students, *F* (2, 405) = 4.458, *p* = 0.012, with no significant difference between the two active groups. This pattern is descriptively consistent with the sports-science literature on ergogenic caffeine use, although caffeine source, dose, and motivation were not assessed; the ergogenic interpretation is therefore offered as a candidate explanation rather than a tested mechanism.

Caffeine is one of the most extensively studied and widely used ergogenic aids in sport and exercise contexts. A robust body of evidence demonstrates that caffeine supplementation (3–6 mg/kg body mass) enhances endurance performance, attenuates perceived exertion, and delays neuromuscular fatigue (28, 46). Physically active university students are increasingly embedded in a performance-oriented exercise culture that normalises pre-workout consumption of caffeinated products, including energy drinks, pre-workout formulations, and caffeine tablets (18, 21, 28, 47, 48). Habitual ergogenic caffeine use may progressively generate the tolerance, withdrawal, and compulsive use patterns that the CUDQ specifically assesses (1, 5, 20), a mechanism that would account for the elevated disorder scores observed in active relative to inactive individuals; however, given the cross-sectional design and the absence of caffeine-motivation data, this pathway remains speculative and should be tested directly in future research. Whether the absence of a significant difference between the minimally active and sufficiently active groups reflects a non-linear, possibly threshold-shaped relationship between habitual PA and CUD severity is an empirical question for future work; no formal non-linear test was conducted here, and the pattern is therefore offered as an exploratory hypothesis rather than as a finding.

### Moderating role of PA in the SMA-CUD relationship

Despite the main-effect finding that PA group membership was associated with differential CUD scores in the ANOVA, hierarchical moderated regression, in which PA was operationalised continuously (MET-min/week) to preserve statistical power and metric precision, revealed that the PA × SMA interaction term did not significantly improve model fit (*β* = 0.043, *p* = 0.358; Δ*R*^2^ = 0.002). The direct predictive effect of SMA on CUD remained robust (*β* = 0.373, *p* < 0.001), confirming the stability of this association across all PA levels and after controlling for sex. Categorical discretisation of the continuous PA variable in the ANOVA reduces statistical power, rendering the regression framework the more appropriate vehicle for the moderation question (36); the regression finding is therefore treated as the primary test of this hypothesis. The apparent discrepancy between the significant ANOVA effect of PA group on CUD and the non-significant continuous PA predictor in the regression cannot be resolved within the present design. One candidate explanation is a non-linear, possibly threshold-shaped relationship in which inactive students differ categorically from both active groups while no linear dose–response gradient operates across the PA continuum; however, this hypothesis was not formally tested and is offered only as a target for future polynomial, piecewise, or SEM-based modelling.

A parsimonious interpretation of the null moderation is therefore adopted: PA, as operationalised here, simply did not modify the SMA-CUD association in this sample. Under the I-PACE-informed framework, this finding is consistent with, though it does not confirm, the possibility that the link is driven primarily by person-level variables (e.g., impulsivity, reward sensitivity, emotion dysregulation) that operate independently of habitual activity level (9, 49). Further mechanistic elaboration of an effect of Δ*R*^2^ = 0.002 is deliberately avoided, since such elaboration would risk post-hoc rationalisation. Practically, the result implies only that PA promotion alone should not be expected to disrupt the PSMU-CUD coupling; replication in larger and more balanced samples, together with direct measurement of the candidate person-level variables, is required before any intervention recommendation can be drawn.

### Limitations

Several limitations qualify the present findings. First, the cross-sectional design precludes causal inference: the direction and temporal sequencing of the SMA-CUD relationship cannot be established from these data; longitudinal designs are necessary to map developmental trajectories and to determine whether SMA prospectively predicts CUD onset, CUD reinforces SMA, or a common third variable drives both patterns. Second, although multivariate outlier screening (Mahalanobis distance) and listwise deletion followed standard practice, the exclusion of 33 participants (7.5%) raises the possibility that the retained sample differs systematically from those excluded, potentially attenuating true variance and underrepresenting students with the most severe profiles. Third, PA was measured via self-report (IPAQ-SF), which is susceptible to recall and social desirability biases; objective accelerometry would improve measurement precision. It should further be noted that MET-based classification thresholds carry inherent measurement error when computed from recall-based data, which may affect the precision of group-level PA classifications. Furthermore, the IPAQ-SF scoring protocol applied here counts only activity bouts of at least 10 consecutive minutes, consistent with the original validation guidelines (35); the WHO (50) physical activity guidelines subsequently removed this minimum-bout requirement, meaning that the present procedure may systematically underestimate total PA volume in individuals who accumulate activity in shorter, interrupted episodes, a caveat of particular relevance when interpreting group-level classifications. Fourth, participants were recruited through convenience sampling via an online survey, which introduces selection bias; students with higher digital engagement may have been disproportionately represented, potentially inflating SMA scores; future studies should employ probability-based sampling. Fifth, the CUDQ does not distinguish caffeine sources or use motivations (alertness, academic performance, ergogenic enhancement, habit), distinctions critical to interpreting the PA-CUD relationship and the ergogenic mechanism proposed here; additionally, the CUDQ’s retrospective 12-month recall window is itself a potential source of measurement artefact, as participants may inaccurately report the frequency or severity of symptoms experienced over this extended period. Sixth, the BSMAS’s six-item brevity does not capture platform-specific behaviour, content type, or passive versus active engagement; these distinctions are theoretically relevant to the dopaminergic reward hypothesis (4) and their absence constrains the mechanistic interpretability of the SMA construct as operationalised here. Seventh, no data were collected on potential third-variable confounds, such as sleep duration, academic workload, and chronotype, that could independently drive both SMA and CUD and thus partially account for their observed co-occurrence; future studies should include these covariates to disambiguate shared-mechanism from common-cause explanations. Finally, the sample is exclusively Turkish, limiting cross-cultural generalisability; replication in culturally diverse settings with varying caffeine norms and digital media landscapes is warranted (21). Additionally, the BSMAS total score threshold of ≥ 18 applied in descriptive comparisons lacks validated clinical referent status, which constrains diagnostic interpretability and precludes clinical-level prevalence estimation from the present data. Three further limitations follow directly from points raised at peer review. Online convenience sampling almost certainly over-represents heavier social-media users, which may inflate mean BSMAS scores and partially attenuate the observed BSMAS-CUDQ correlation through range restriction; probability-based or stratified recruitment in future replications would mitigate this bias. The inactive subgroup was severely underpowered (n = 23, 5.6%), which limits the precision of all PA-group comparisons and especially the inactive vs. active contrasts on CUD; balanced recruitment across PA strata, or oversampling of inactive students, is needed to interpret the apparent CUD-by-PA effect with confidence. The discrepancy between the significant categorical PA effect on CUD (ANOVA) and the non-significant continuous PA term (regression) is consistent with a non-linear, possibly threshold-shaped relationship, but no formal non-linear analysis was conducted here. Future work should test this directly using polynomial (e.g., quadratic) PA terms, piecewise/spline regression, or a structural-equation framework that accommodates non-linear paths and latent indicators of PSMU and CUD; the present results should therefore be regarded as supporting a prima facie case for further non-linear modelling rather than as a definitive linear test.

## Conclusion

Within the limits of a single cross-sectional study with modest effect sizes, the present analyses examined PSMU, CUD, and PA in university students under a unified analytical framework, and four tentative observations follow.

First, PSMU and CUD showed a statistically significant, small-to-moderate positive association in this sample. Joint screening of these two patterns within university health services may be worth pilot evaluation, but should not be implemented as routine practice on the basis of cross-sectional findings alone. Second, PA level did not differentiate students on SMA severity. This null finding is most parsimoniously read as PSMU and habitual PA being largely independent in this sample; no intervention recommendation is drawn from a near-zero effect (*η*^2^p = 0.001). Third, active students reported higher CUD scores than inactive students, a descriptive pattern compatible with, but not a direct test of, the ergogenic caffeine use hypothesis; the small inactive subsample (*n* = 23) further restricts the strength of this comparison and any educational recommendation built on it. Fourth, no moderation by PA was detected (Δ*R*^2^ = 0.002); the simplest reading is that, in this sample, PA does not modify the PSMU-CUD association.

Taken together, the present cross-sectional findings provide preliminary evidence that PSMU and CUD co-occur to a small-to-moderate degree among Turkish university students, that habitual PA does not appear to moderate this association, and that mean CUD scores are higher in active than inactive participants in a pattern descriptively compatible with ergogenic caffeine use. Stronger inferences and any prevention or intervention recommendation await longitudinal, multi-method, and cross-cultural replication that directly tests the candidate mechanisms (sleep disruption, transdiagnostic person variables, ergogenic caffeine motives) discussed here, ideally with balanced PA strata and formal modelling of non-linear effects.

## Data Availability

The raw data supporting the conclusions of this article will be made available by the authors, without undue reservation.
